# Supplementary material for: Optimal microRNA Sequencing Depth to Predict Cancer Patient Survival with Random Forest and Cox Models
Source: Genes (Basel). 2022 Dec 2;13(12):2275. doi: 10.3390/genes13122275 (PMC9777708; doi:10.3390/genes13122275)
Supplement: Supplementary file 1 [file genes-13-02275-s001.zip › genes-2026736-supplementary.pdf]

Supplementary Materials

1 More details on the Cox model

The function L is called the 'pseudo-likelihood', because it is not a product of density functions, but a product of conditional probabilities. $\hat{\boldsymbol{\beta}}$ is computed by maximizing this pseudo-likelihood function: $\hat{\boldsymbol{\beta}} = \arg\max_{\boldsymbol{\beta}}(l(\boldsymbol{\beta}))$, with $l(\boldsymbol{\beta}) = \log(L(\boldsymbol{\beta}))$, the log-pseudo-likelihood.

Note that the Cox model is not intuitive, in the sense that it links genetic data to patient survival in an indirect way, through the hazard function. However, Cox pseudo-likelihood allows censored data to be efficiently dealt with. Moreover, this yields a robust inference procedure where the baseline function $h_0(t)$ does not need to be modeled or estimated in a parametric way. Finally, the estimation procedure leads to a convex optimization problem, for which efficient procedures and packages exist for computing $\hat{\boldsymbol{\beta}}$ [Friedman et al., 2010].

2 More details on the penalization methods

The ℓ_1 norm forces some coefficient estimates $\hat{\beta}_j, j = 1, ..., p$ to be zero, and allows the selection to be made. For multivariate Cox selection models, the genes selected are defined as the genes with nonzero $\hat{\beta}_j$ coefficients. It has been empirically observed that if there are high correlations between predictors, the ridge penalty provides better prediction performance than the lasso [Tibshirani, 1997]. The elastic net penalty have been developed to tackle this issue.

We computed the weight of the penalty, λ, by K-fold cross-validation (K = 5) using the R package *glmnet* [Friedman et al., 2010]. The weight λ that minimizes deviation in the cross-validation is given by λ_{min}. We chose $\alpha = 0.3$ in the elastic net, as the deviance remains stable for different values of α and the number of genes selected starts to stabilize below this value (Supplementary Fig. S1).

For more details of the mathematical concepts used in this article, we refer the reader to the book 'The Statistical Analysis of Failure Time Data' [Kalbfleisch and Prentice, 2011].

References

[Friedman et al., 2010] Friedman, J. et al. (2010). Regularization paths for generalized linear models via coordinate descent. *Journal of Statistical Software*, 33(1):1–22.

[Kalbfleisch and Prentice, 2011] Kalbfleisch, J. D. and Prentice, R. L. (2011). *The Statistical Analysis of Failure Time Data*. AMLBook.

[Tibshirani, 1997] Tibshirani, R. (1997). The lasso method for variable selection in the cox model. *Statistics in Medicine*, 16(4):385–395.

Supplementary Tables

Cancer	Name
LAML	Acute Myeloid Leukemia
ACC	Adrenocortical carcinoma
BLCA	Bladder Urothelial Carcinoma
LGG	Brain Lower Grade Glioma
BRCA	Breast invasive carcinoma
CESC	Cervical squamous cell carcinoma and endocervical adenocarcinoma
CHOL	Cholangiocarcinoma
LCML	Chronic Myelogenous Leukemia
COAD	Colon adenocarcinoma
ESCA	Esophageal carcinoma
GBM	Glioblastoma multiforme
HNSC	Head and Neck squamous cell carcinoma
KICH	Kidney Chromophobe
KIRC	Kidney renal clear cell carcinoma
KIRP	Kidney renal papillary cell carcinoma
LIHC	Liver hepatocellular carcinoma
LUAD	Lung adenocarcinoma
LUSC	Lung squamous cell carcinoma
DLBC	Lymphoid Neoplasm Diffuse Large B-cell Lymphoma
MESO	Mesothelioma
MISC	Miscellaneous
OV	Ovarian serous cystadenocarcinoma
PAAD	Pancreatic adenocarcinoma
PCPG	Pheochromocytoma and Paraganglioma
PRAD	Prostate adenocarcinoma
READ	Rectum adenocarcinoma
SARC	Sarcoma
SKCM	Skin Cutaneous Melanoma
STAD	Stomach adenocarcinoma
TGCT	Testicular Germ Cell Tumors
THYM	Thymoma
THCA	Thyroid carcinoma
UCS	Uterine Carcinosarcoma
UCEC	Uterine Corpus Endometrial Carcinoma
UVM	Uveal Melanoma

Supplementary Tab. S1. Acronym of the TCGA cancers

cancer	median miRNA sequencing depth	Number of patients
**ACC**	5 119 430	77
**CESC**	4 692 352	296
**KIRC**	2 502 855	528
**KIRP**	6 049 207	271
**LGG**	7 452 065	514
**LIHC**	4 784 378	363
**LUAD**	4 865 507	492
**MESO**	5 170 703	87
**PRAD**	4 292 594	490
**UCEC**	4 302 391	542
**UVM**	4 598 102	77
BLCA	4 838 386	373
BRCA	2 908 082	1 093
COAD	3 589 624	448
ESCA	3 951 954	167
HNSC	4 609 615	491
LAML	730 327	173
LUSC	3 442 320	488
OV	3 818 682	301
PAAD	5 068 328	176
READ	3 787 196	163
STAD	3 699 767	409
TGCT	5 305 326	124
THCA	5 505 326	500
THYM	5 839 688	119

Supplementary Tab. S2. Median microRNA sequencing depth and number of patients in the TCGA cohort. The retained 11 cancers for further analysis are indicated first, in bold.

A

Cancer	UVM	ACC	KIRP	MESO	KIRC	LGG	CESC	LIHC	PRAD	LUAD	UCEC
Fold reduction	100	10	5	5	10	10	1000	100	10	10	100
Corresponding median sequencing depth	50	500	1000	1000	200	700	5	50	400	500	40
Metric degraded first	C-index	C-index	C-index	both	both	IBS	C-index	both	C-index	C-index	C-index

B

Cancer	UVM	ACC	KIRP	MESO	KIRC	LGG	CESC	LIHC	PRAD	LUAD	UCEC
Fold reduction	100	100	100	100	1000	1000	100	100	100	100	100
Corresponding median sequencing depth	400	400	400	500	50	50	500	500	500	400	200
Metric degraded first	IBS	both	C-index	both	both	both	C-index	C-index	C-index	C-index	C-index

Supplementary Tab. S3. Maximum fold reduction without degradation of the C-index and the IBS, corresponding median sequencing depth (thousands of reads), and prediction metric degraded first for random survival forest and for miRNA-seq data (A) and mRNA-seq data (B) for the 11 investigated cancers.

A miRNA - Cox

Cancer	UVM	ACC	KIRP	MESO	KIRC	LGG	CESC	LIHC	PRAD	LUAD	UCEC
Proportion of patients in the training set	0.4	0.6	0.6	0.6	0.7	0.7	0.6	0.7	0.8	0.8	0.4
Corresponding number of patients	31	46	161	51	356	354	173	248	389	386	213

B miRNA – random survival forest

Cancer	UVM	ACC	KIRP	MESO	KIRC	LGG	CESC	LIHC	PRAD	LUAD	UCEC
Proportion of patients in the training set	0.3	0.4	0.2	0.5	0.4	0.5	0.1	0.5	0.3	0.5	0.2
Corresponding number of patients	23	31	54	42	203	253	29	178	146	242	106

C mRNA - Cox

Cancer	UVM	ACC	KIRP	MESO	KIRC	LGG	CESC	LIHC	PRAD	LUAD	UCEC
Proportion of patients in the training set	0.8	0.5	0.6	0.7	0.6	0.6	0.8	0.7	0.8	0.8	0.7
Corresponding number of patients	62	38	161	59	305	304	230	248	389	386	372

D mRNA – random survival forest

Cancer	UVM	ACC	KIRP	MESO	KIRC	LGG	CESC	LIHC	PRAD	LUAD	UCEC
Proportion of patients in the training set	0.5	0.5	0.3	0.5	0.3	0.3	0.4	0.3	0.2	0.5	0.3
Corresponding number of patients	38	38	81	42	152	152	115	106	97	242	160

Supplementary Tab. S4. Minimum Proportion of patients needed in the training dataset without degradation of the C-index and the IBS and corresponding number of patients for the 11 investigated cancers for miRNA-seq data and the Cox model (A), miRNA-seq data and random survival forest (B), mRNA-seq data and the Cox model (C), and mRNA-seq data and random survival forest (D).

Supplementary Figures

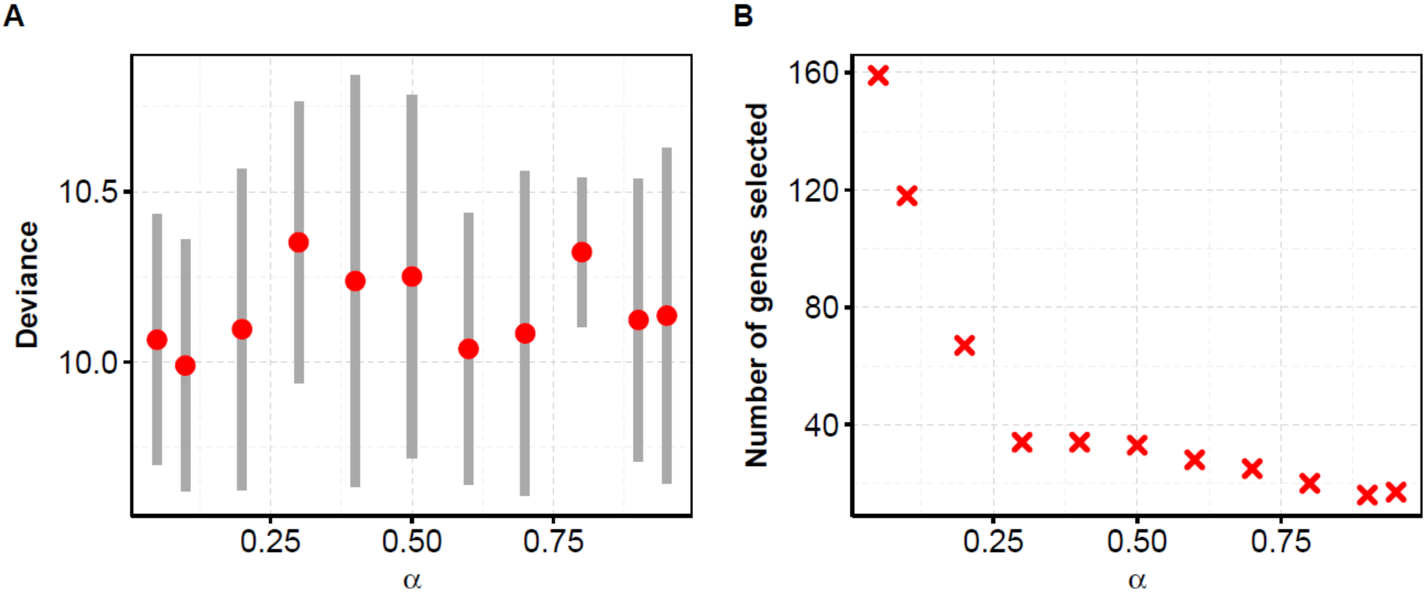

Supplementary Fig. S1. Deviance and number of genes selected for different values of α for KIRP.

We computed the deviance by K-fold cross validation (K=5) for each value of α. Similar behavior is observed for the other cancers (data not shown).

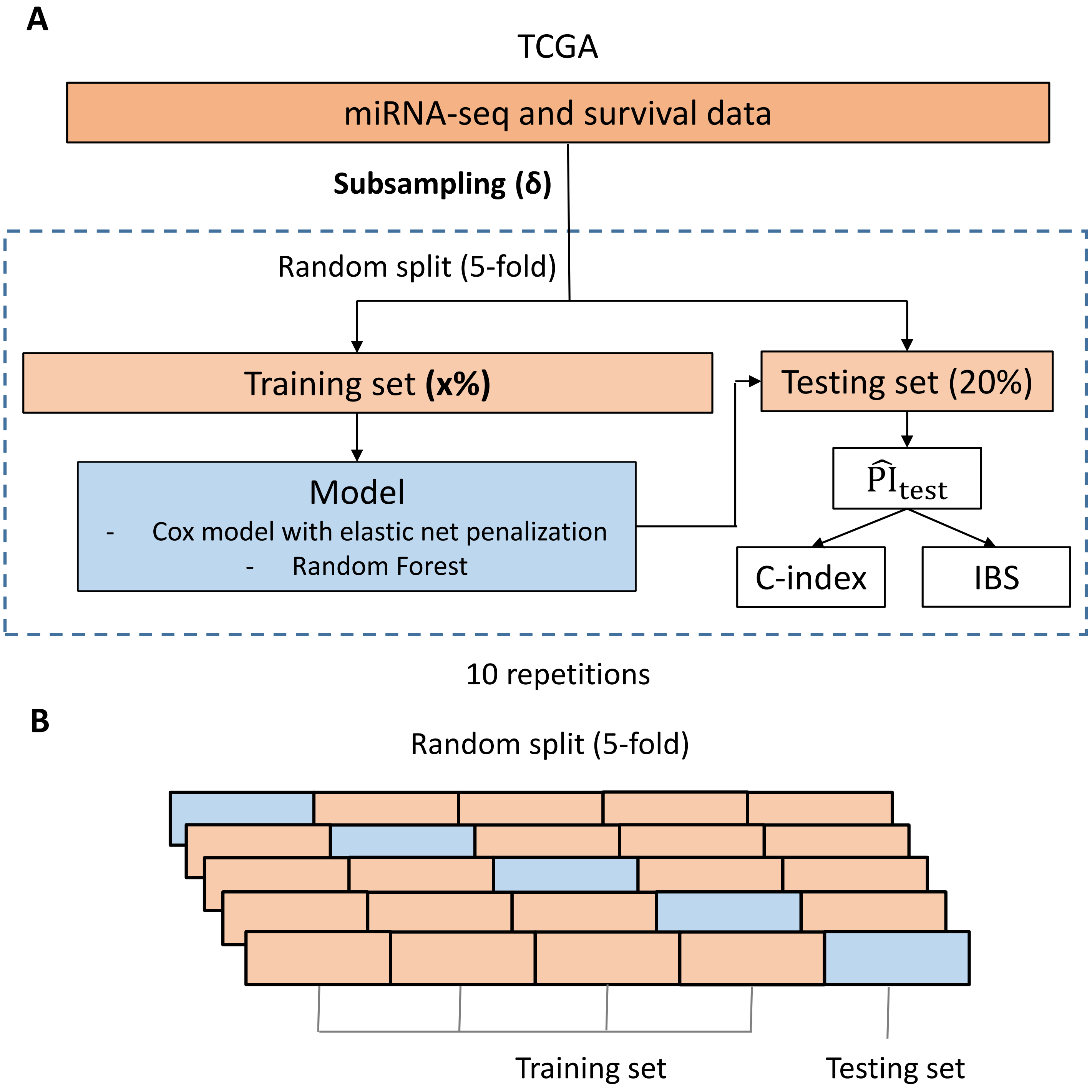

Supplementary Fig. S2. Procedure for the evaluation of prediction performances.

CPM corresponds to Count Per Million normalization, RS means `Risk Score' and IBS refers to integrated Brier score.

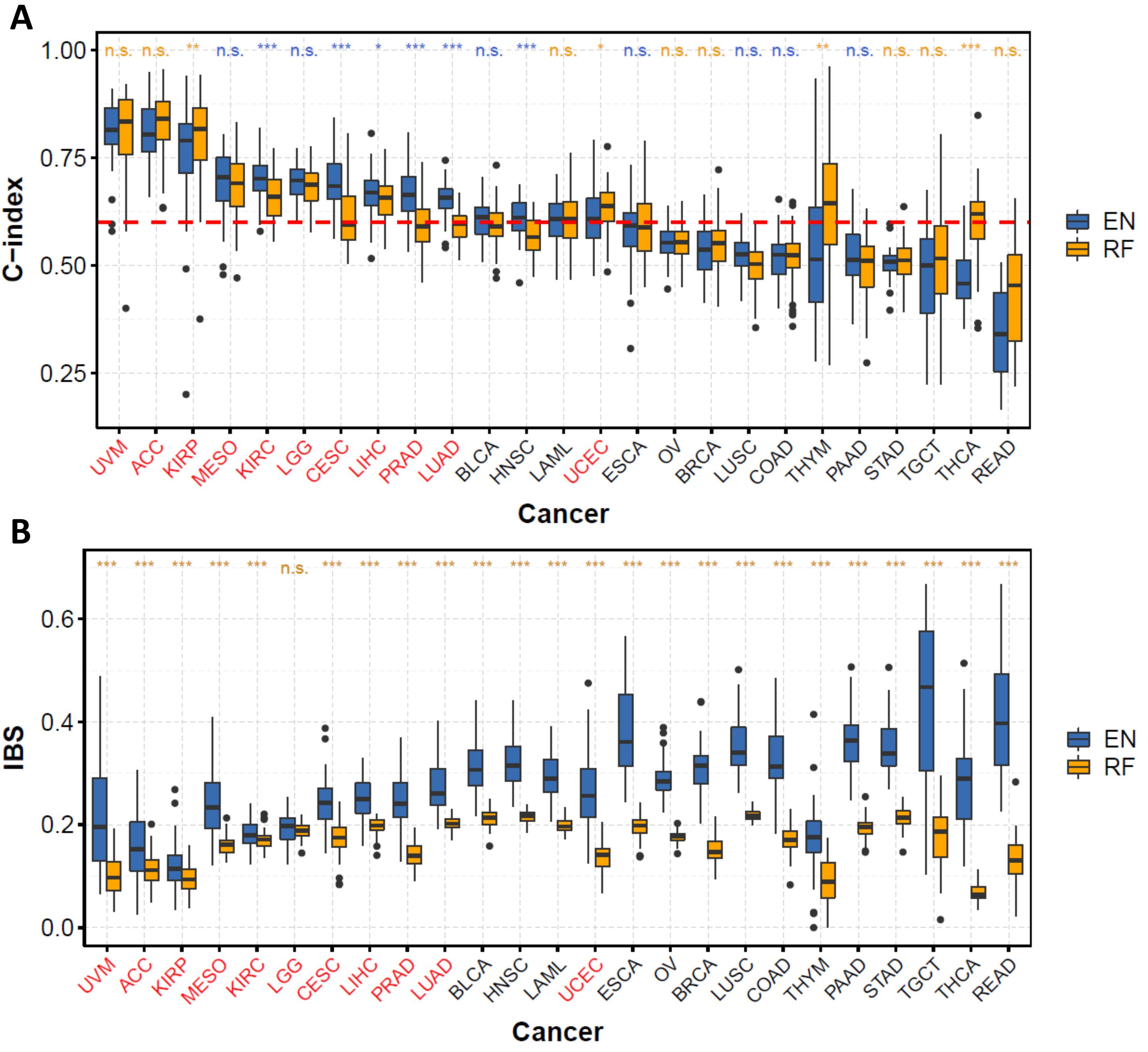

Supplementary Fig. S3. Boxplot of the C-indices (A) and of the IBS (B) for the Cox model with elastic net penalty (blue) and random forest (orange).

We computed the metrics by 10 repetitions of a K-fold cross validation (K=5) for all the 25 cancers. We retained 11 cancers (red) that have a median C-index significantly above 0.6 according to a one-sided Wilcoxon test at level 0.05. We corrected the p-values with the Benjamini-Hochberg method.

To compare the predictions obtained with Cox model and random forest, we did a two-sided wilcoxon signed-rank test between C-indices (resp. IBS). Significance level are above each graphics (blue : median C-index is higher or IBS is lower for the Cox model, orange : median C-index is higher or IBS is lower for random forest).

Red dotted horizontal line : C-index of 0.6.

***: p ≤ 0.001, **: p ≤ 0.01, *: p ≤ 0.05, +: p ≤ 0.1, n.s. : p > 0.1

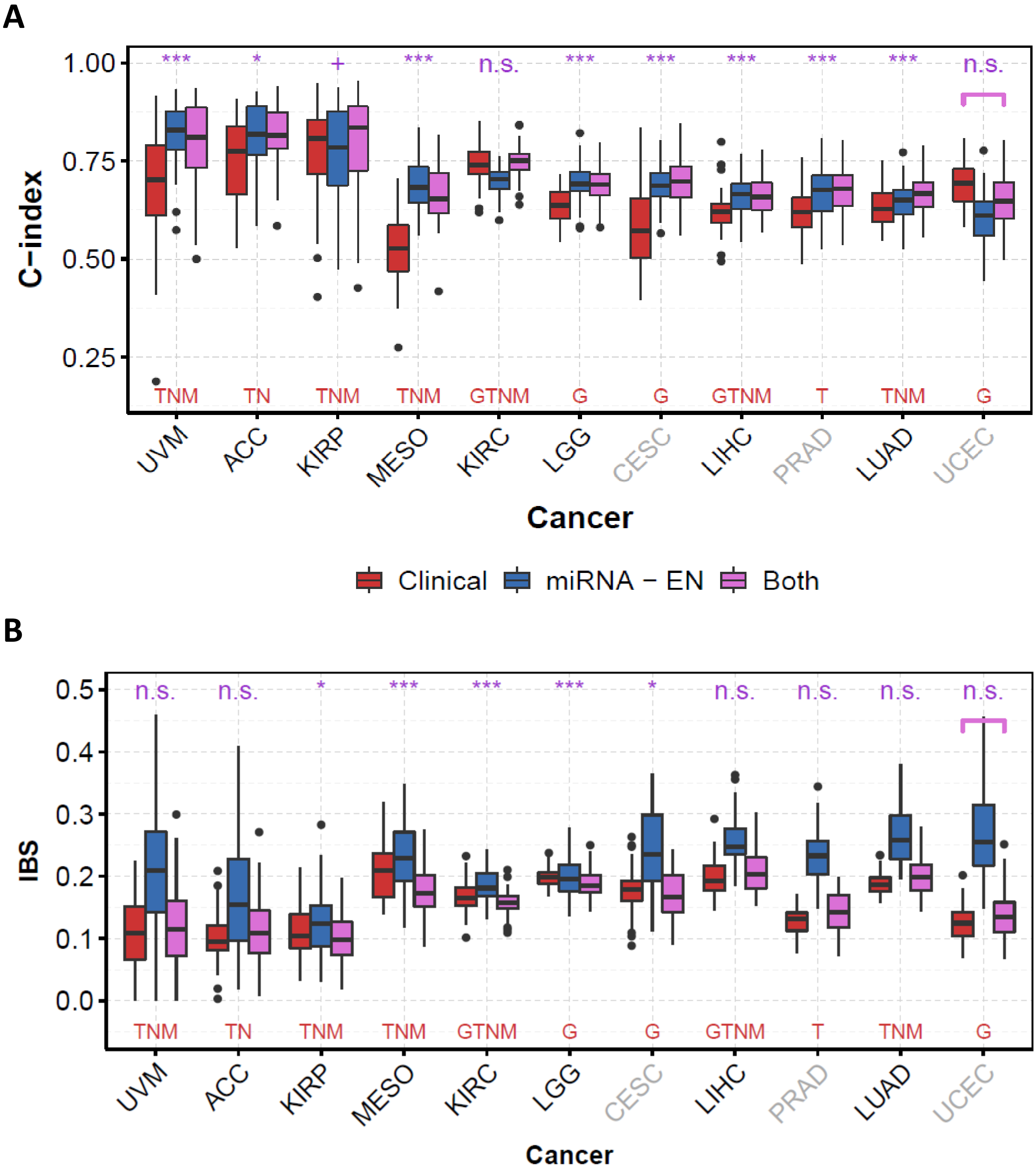

Supplementary Fig. S4. C-indices (A) and IBS (B) obtained with clinical data alone (red), miRNA-seq data alone (blue), and both clinical and miRNA-seq data (purple) for the 11 cancers investigated and the Cox model with elastic net penalty.

We computed the metrics by 10 repetitions of a K-fold cross-validation (K=5). We computed p-values of a one-sided Wilcoxon signed-rank test between Clinical and Both Clinical + miRNAs (purple stars at the top of each graphic, Benjamini-Hochberg correction for the 11 p-values). ***: $p \leq 0.001$, **: $p \leq 0.01$, *: $p \leq 0.05$, +: $p \leq 0.1$, n.s. : $p > 0.1$

Red letters at the bottom of each graphics indicate the clinical data available (G: grade; T: tumor; N: node; M: metastasis). Age is available for all cancers, and gender only for non-unisexual cancers (CESC, PRAD, TGCT are sex-specific).

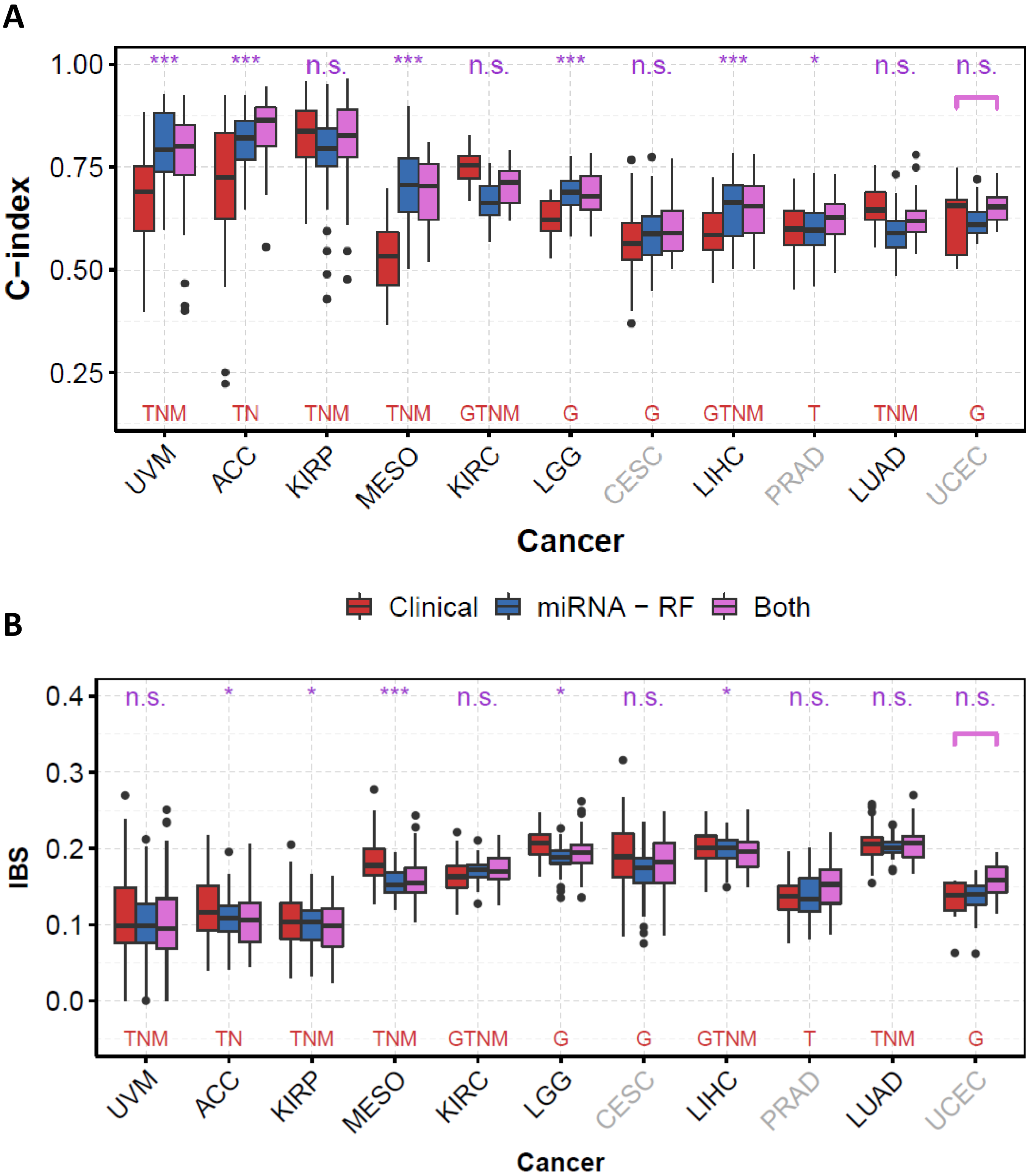

Supplementary Fig. S5. C-indices (A) and IBS (B) obtained with clinical data alone (red), miRNA-seq data alone (blue), and both clinical and miRNA-seq data (purple) for the 11 cancers investigated and the random survival forest procedure.

We computed the metrics by 10 repetitions of a K-fold cross-validation (K=5). We computed p-values of a one-sided Wilcoxon signed-rank test between Clinical and Both Clinical + miRNAs (purple stars at the top of each graphic, Benjamini-Hochberg correction for the 11 p-values). ***: $p \leq 0.001$, **: $p \leq 0.01$, *: $p \leq 0.05$, +: $p \leq 0.1$, n.s. : $p > 0.1$

Red letters at the bottom of each graphics indicate the clinical data available (G: grade; T: tumor; N: node; M: metastasis). Age is available for all cancers, and gender only for non-unisexual cancers (CESC, PRAD, TGCT are sex-specific).

A

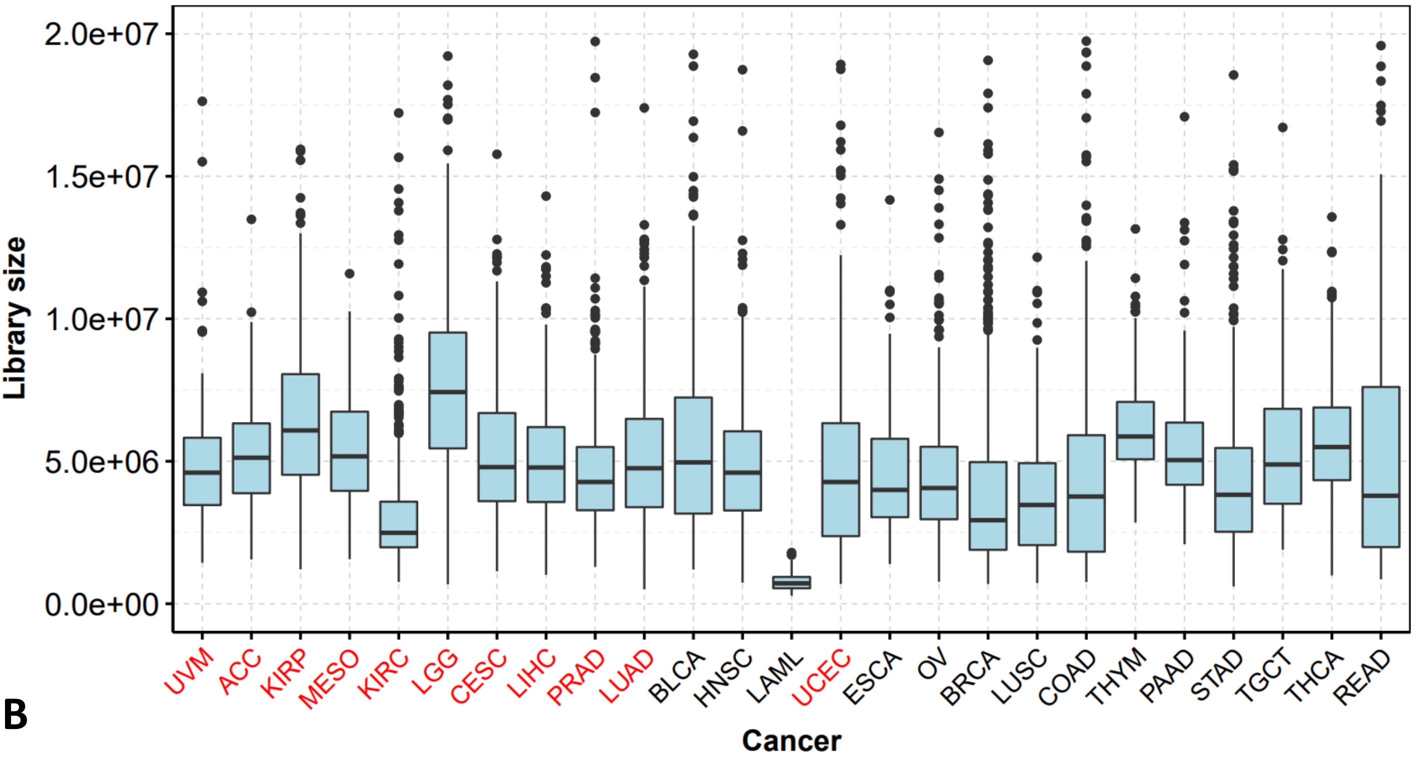

B

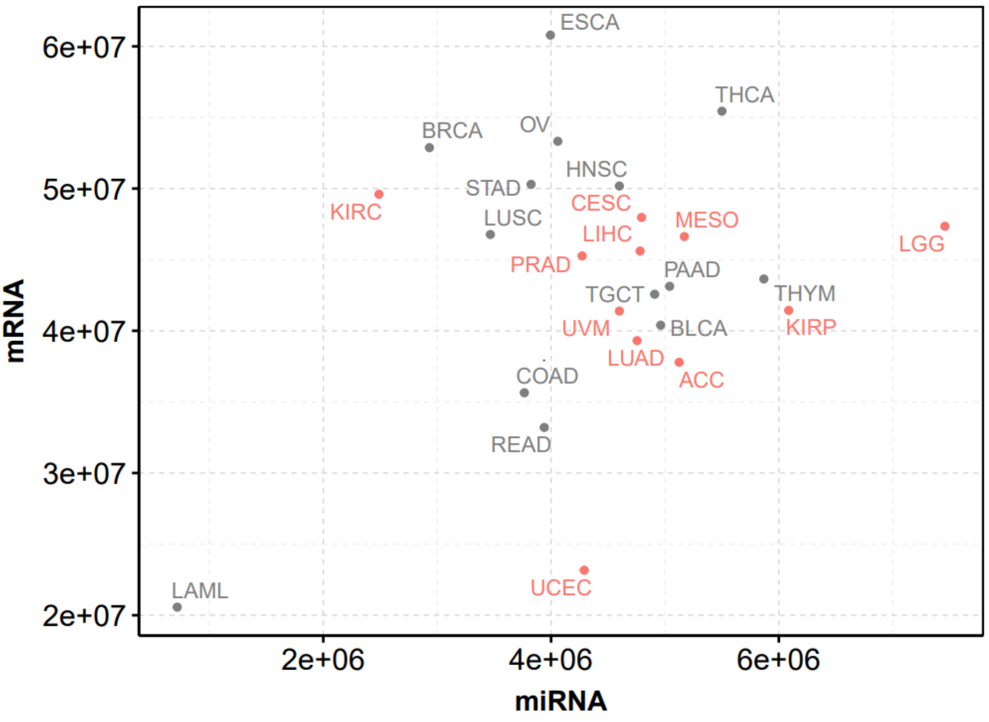

Supplementary Fig. S6. Distribution of the library size for miRNA-seq data (A), and median library size for mRNA-seq and miRNA-seq data(B) for the 25 cancers of TCGA.

The 11 cancers investigated for subsampling are in red.

A

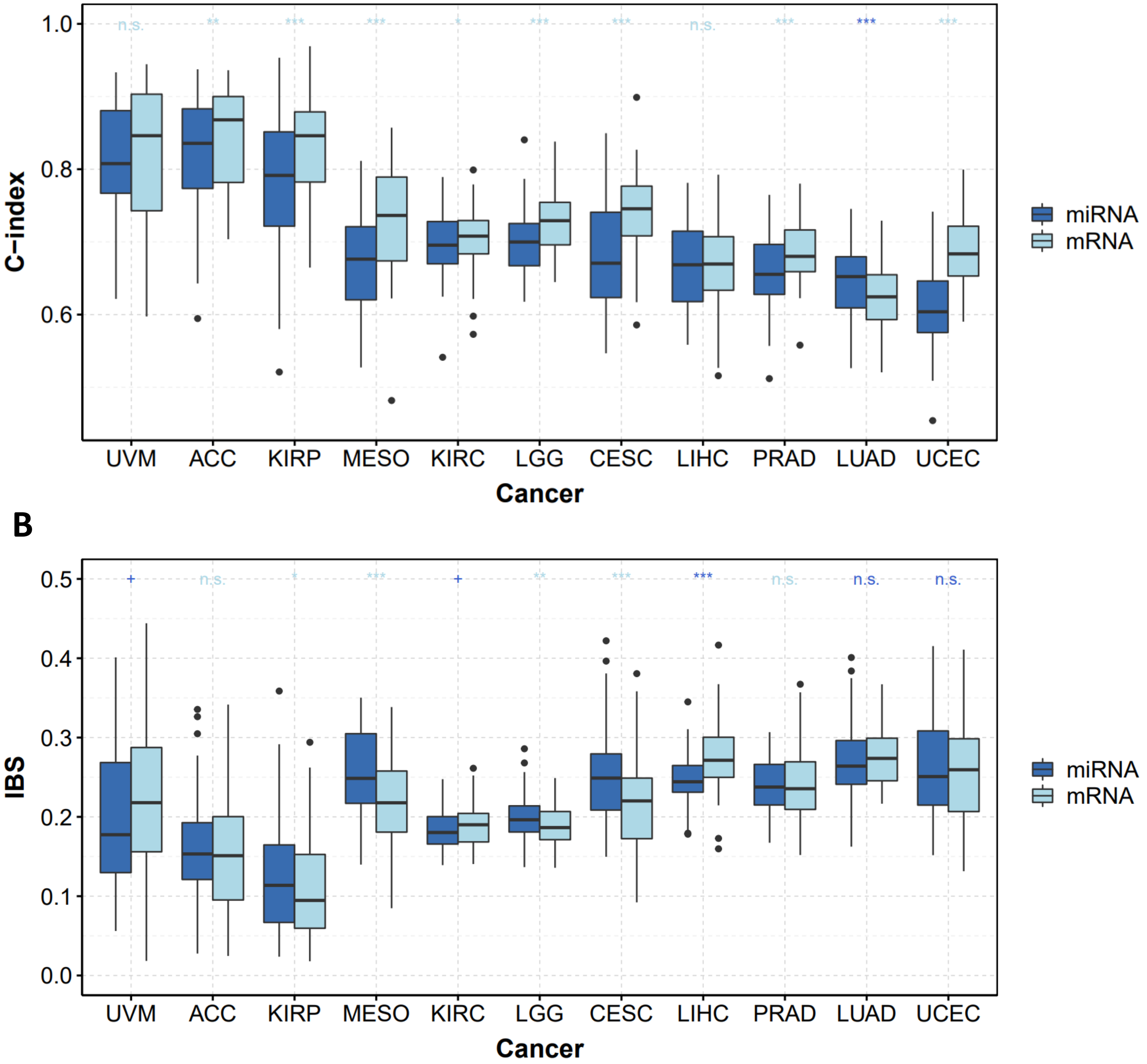

Supplementary Fig. S7. Boxplot of the C-indices (A) and of the IBS (B) for the Cox model with elastic net penalty for miRNA-seq (blue) and mRNA-seq (lightblue) data.

We computed the metrics by 10 repetitions of a K-fold cross validation (K=5) for all the 11 cancers.

To compare the prediction obtained with miRNA-seq and mRNA-seq data, we did a wilcoxon signed-rank test between C-indices (resp. IBS). Significance level with Benjamini-Hocberg correction are above each graphics (blue: median C-index (resp. IBS) is higher (resp. lower) for miRNA-seq data, lightblue: median C-index (resp. IBS) is higher (resp. lower) for mRNA-seq data).

***: p ≤ 0.001, **: p ≤ 0.01, *: p ≤ 0.05, +: p ≤ 0.1, n.s. : p > 0.1

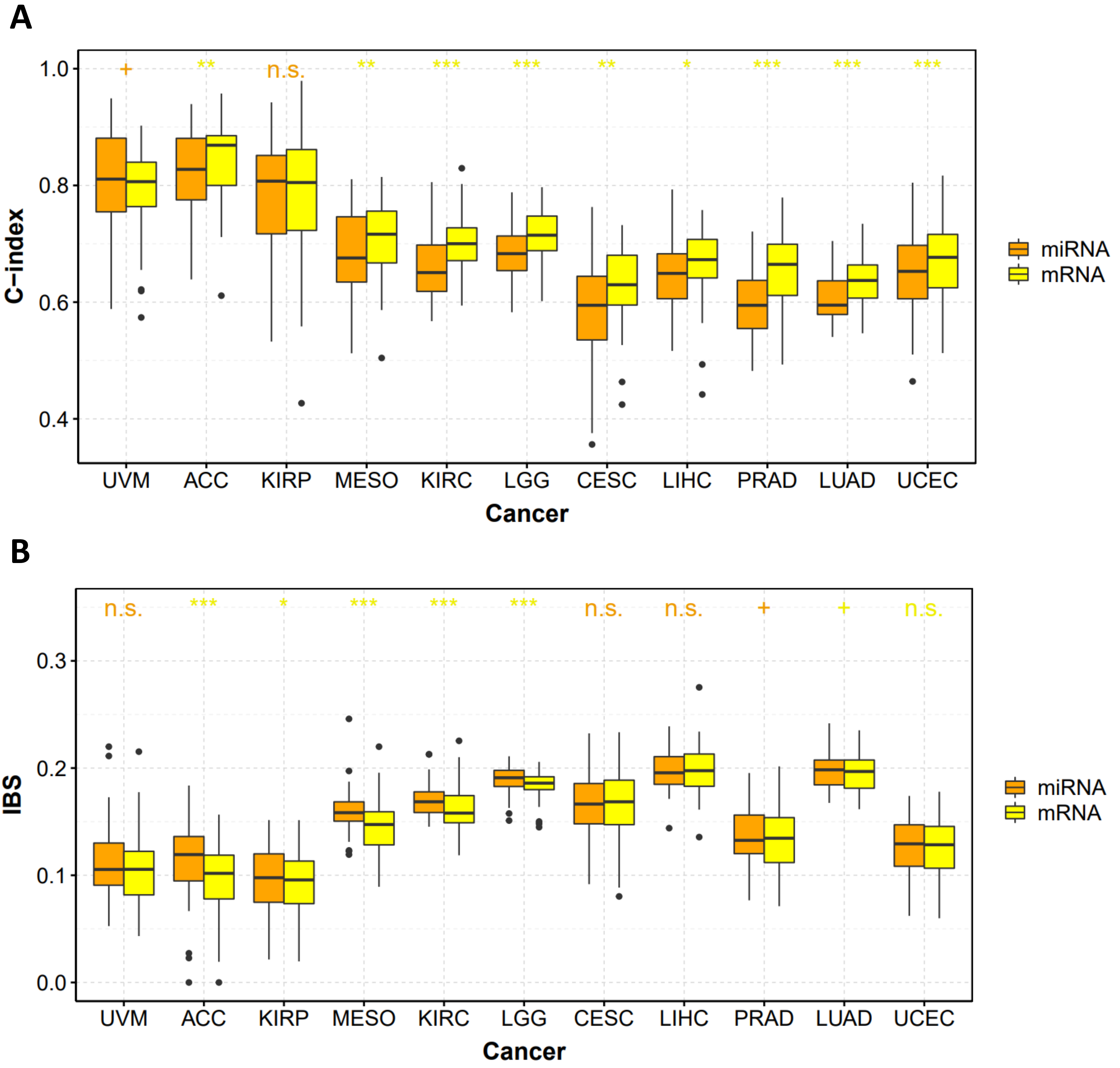

Supplementary Fig. S8. Boxplot of the C-indices (A) and of the IBS (B) for random survival forest for miRNA-seq (orange) and mRNA-seq (yellow) data.

We computed the metrics by 10 repetitions of a K-fold cross validation (K=5) for all the 11 cancers.

To compare the prediction obtained with miRNA-seq and mRNA-seq data, we did a wilcoxon signed-rank test between C-indices (resp. IBS). Significance level with Benjamini-Hocberg correction are above each graphics (orange: median C-index (resp. IBS) is higher (resp. lower) for miRNA-seq data, yellow: median C-index (resp. IBS) is higher (resp. lower) for mRNA-seq data).

***: $p \leq 0.001$, **: $p \leq 0.01$, *: $p \leq 0.05$, +: $p \leq 0.1$, n.s. : $p > 0.1$

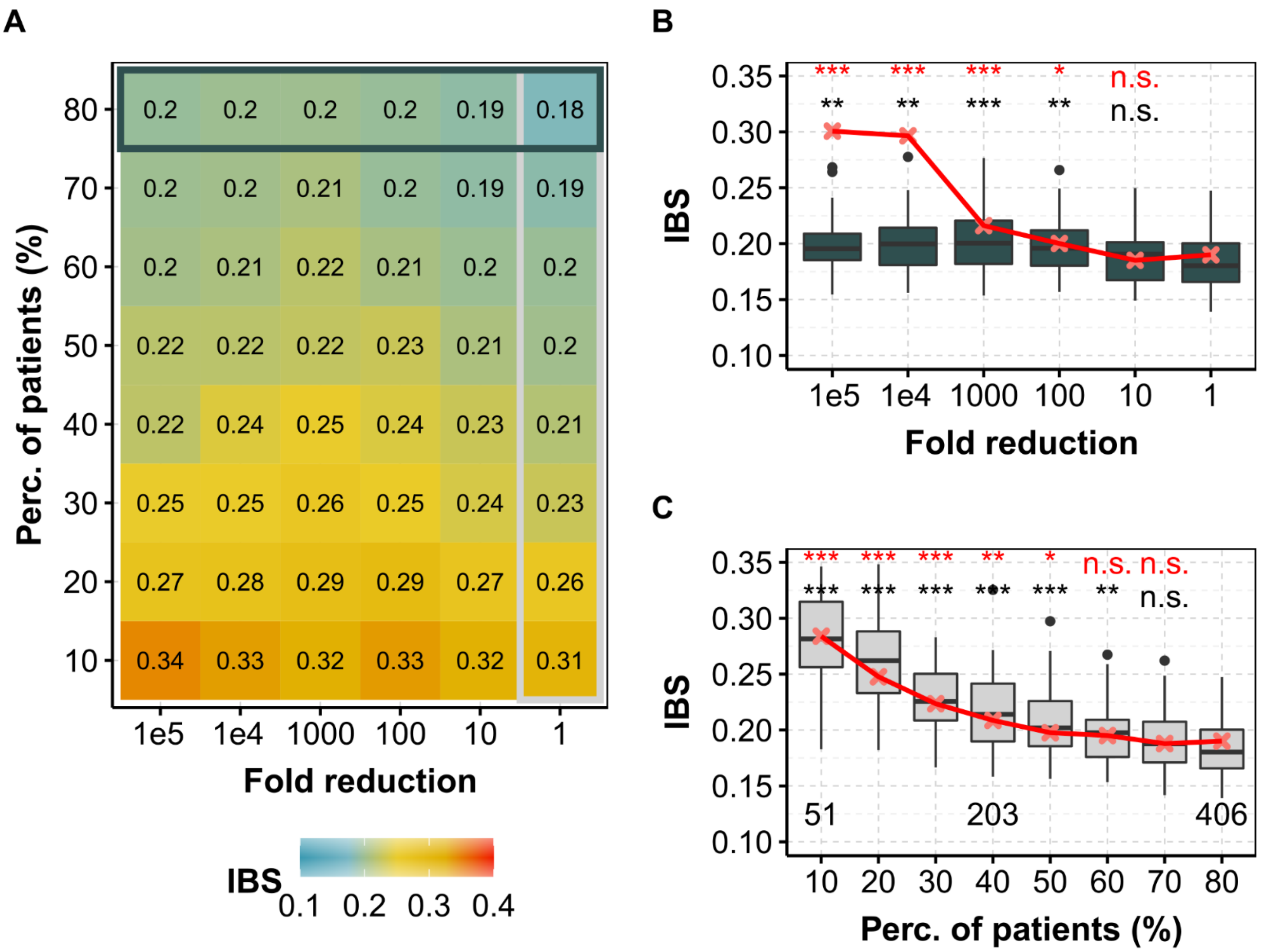

Supplementary Fig. S9. IBS obtained for different fold reduction factors and percentage of patients in the training dataset for KIRC (ccRCC, TCGA) with the Cox model.
Same as Fig. 1 but for IBS.

(A) Median IBS for different degradation of both sequencing depth (x axis) and percentage of patients (y axis) in the training dataset for miRNA-seq data. Horizontal box highlights the case where all of the 80% of patients are used and corresponds to (B), whereas vertical box focuses on the full available library size and corresponds to (C). **(B)** IBS for different fold reduction factors for miRNA-seq (gray boxplots) and mRNA-seq data (median values, in red) with 80% of the patients in the training dataset. Above is the p-value of a one-sided Wilcoxon test compared to no subsampling (*i.e.* $\delta = 1$). **(C)** IBS for different percentage of patients in the training dataset for miRNA-seq (light gray boxplots) and mRNA-seq data (median values, in red) with original TCGA sequencing depth. Above is the p-value of a one-sided Wilcoxon test compared to full dataset (i.e. 80%).
red, mRNA-seq; gray (boxplots), miRNA-seq.

In each case, we computed the IBS by 10 repetitions of a 5-fold cross validation.
***: $p \leq 0.001$, **: $p \leq 0.01$, *: $p \leq 0.05$, +: $p \leq 0.1$, n.s. : $p > 0.1$

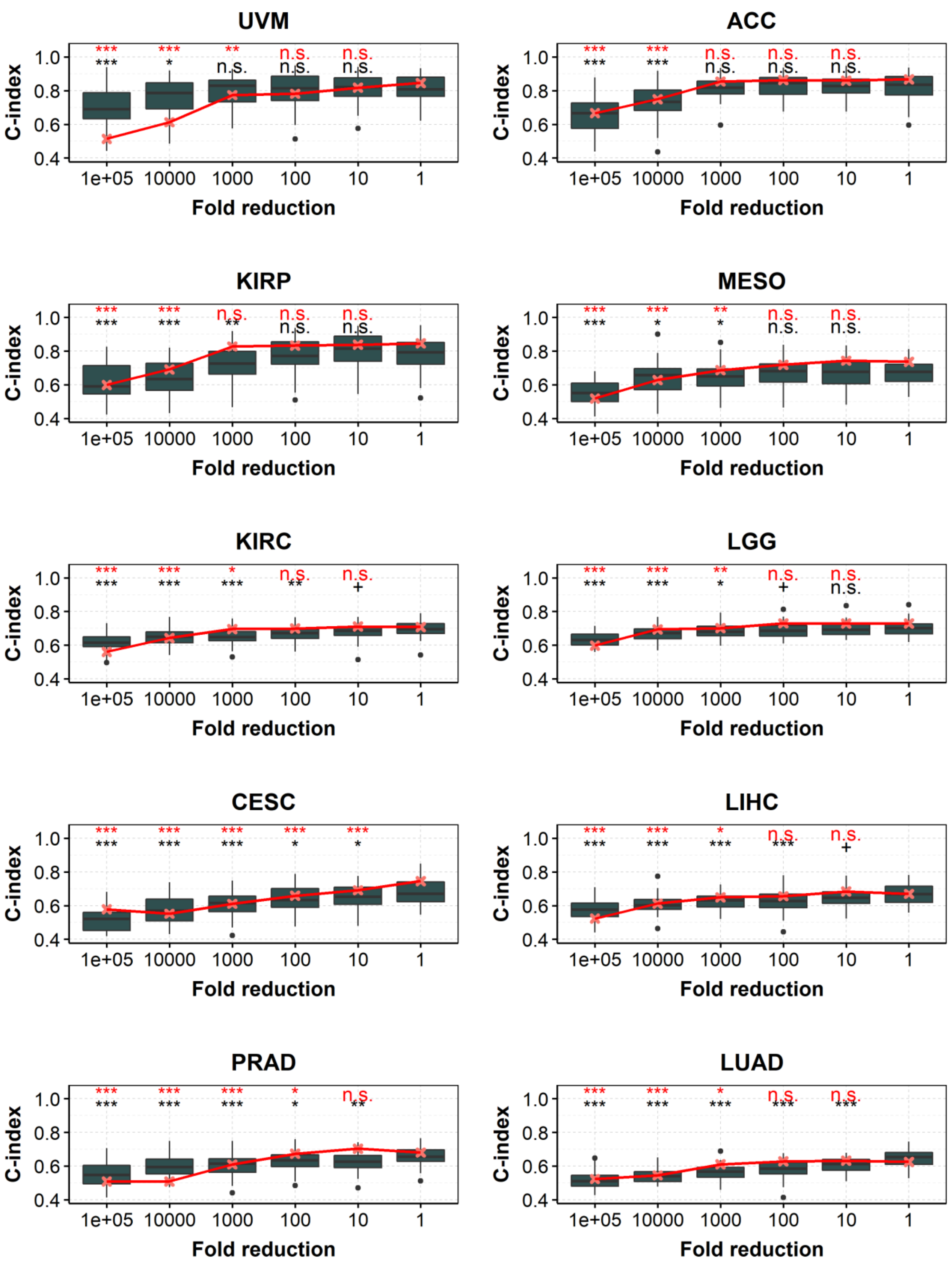

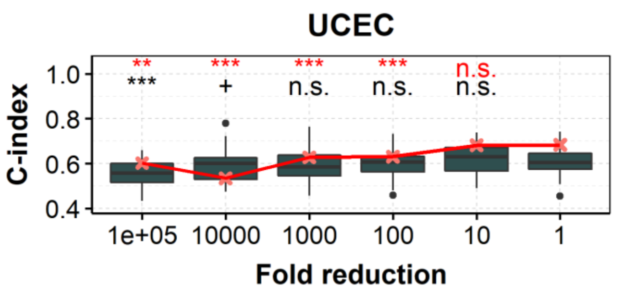

Supplementary Fig. S10. Corresponds to Figure 1B, but for the 11 investigated cancers. Cox model.
Full legend next page.

Supplementary Fig. S10. Distribution of C-indices obtained with the Cox model after fold reduction of miRNA-seq data or mRNA-seq data for the 11 investigated cancers.
C-index for different fold reduction factors for miRNA-seq (gray boxplots) and mRNA-seq data (median values, in red) with 80% of the patients in the training dataset. Above is the pvalue
of a one-sided Wilcoxon test compared to no subsampling (i.e. d = 1). red, mRNA-seq; gray, miRNA-seq
In each case, we computed the C-indices by 10 repetitions of a 5-fold cross validation.
***: p ≤ 0.001, **: p ≤ 0.01, *: p ≤ 0.05, n.s. : p > 0.1.

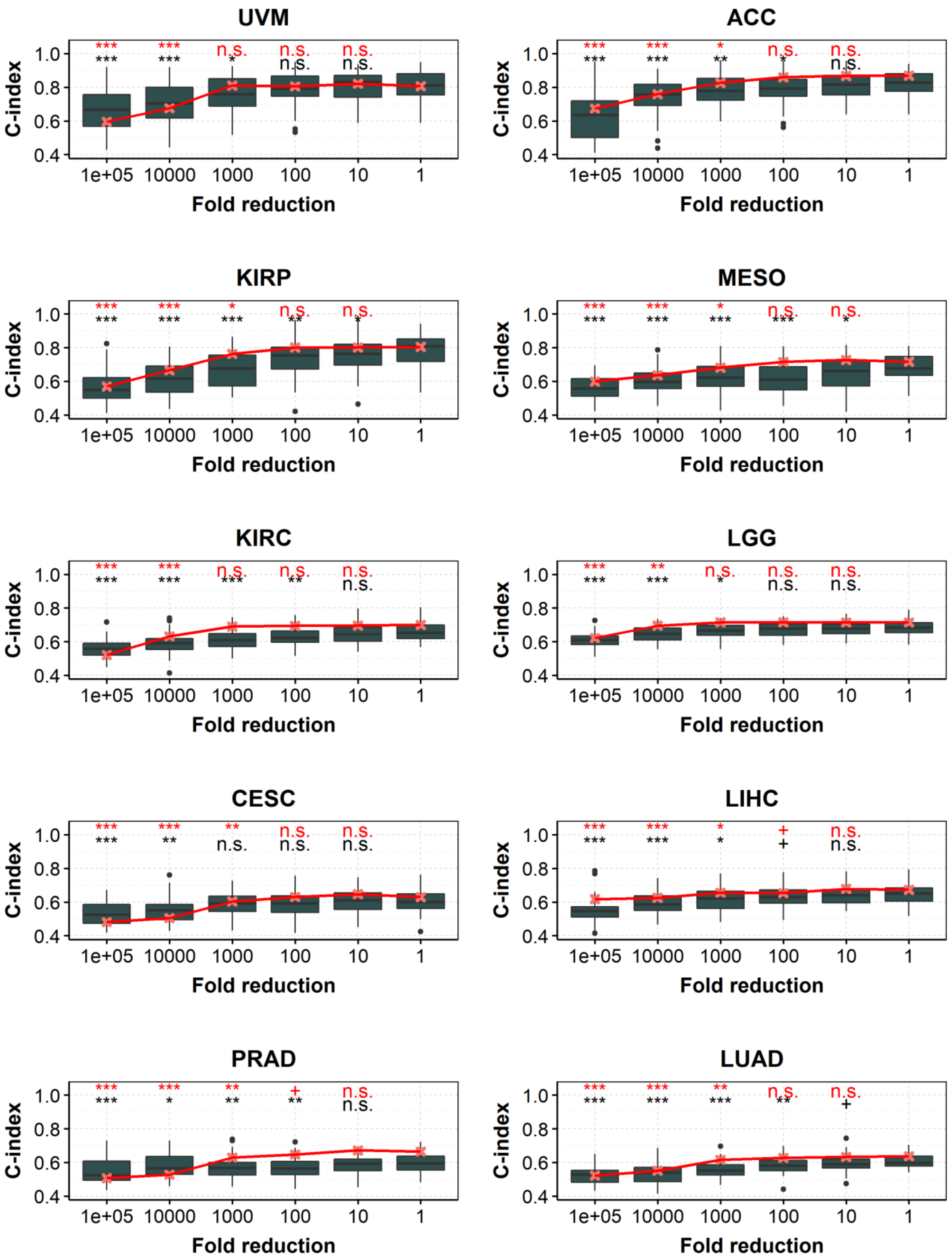

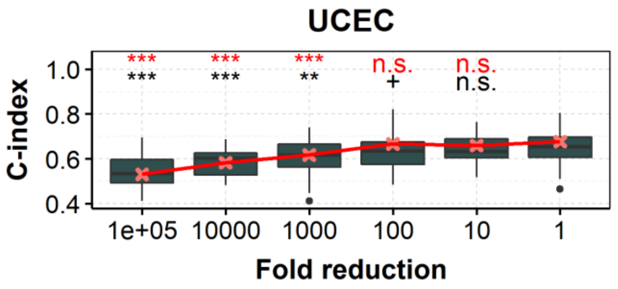

Supplementary Fig. S11. Corresponds to Figure 1B, but for the 11 investigated cancers and for random survival forest.
Full legend next page.

Supplementary Fig. S11. Distribution of C-indices obtained with the random survival forest model after fold reduction of miRNA-seq data or mRNA-seq data for the 11 investigated cancers.

C-index for different fold reduction factors for miRNA-seq (gray boxplots) and mRNA-seq data (median values, in red) with 80% of the patients in the training dataset. Above is the pvalue
of a one-sided Wilcoxon test compared to no subsampling (i.e. d = 1). red, mRNA-seq; gray, miRNA-seq
In each case, we computed the C-indices by 10 repetitions of a 5-fold cross validation.
***: $p \leq 0.001$, **: $p \leq 0.01$, *: $p \leq 0.05$, n.s. : $p > 0.1$.

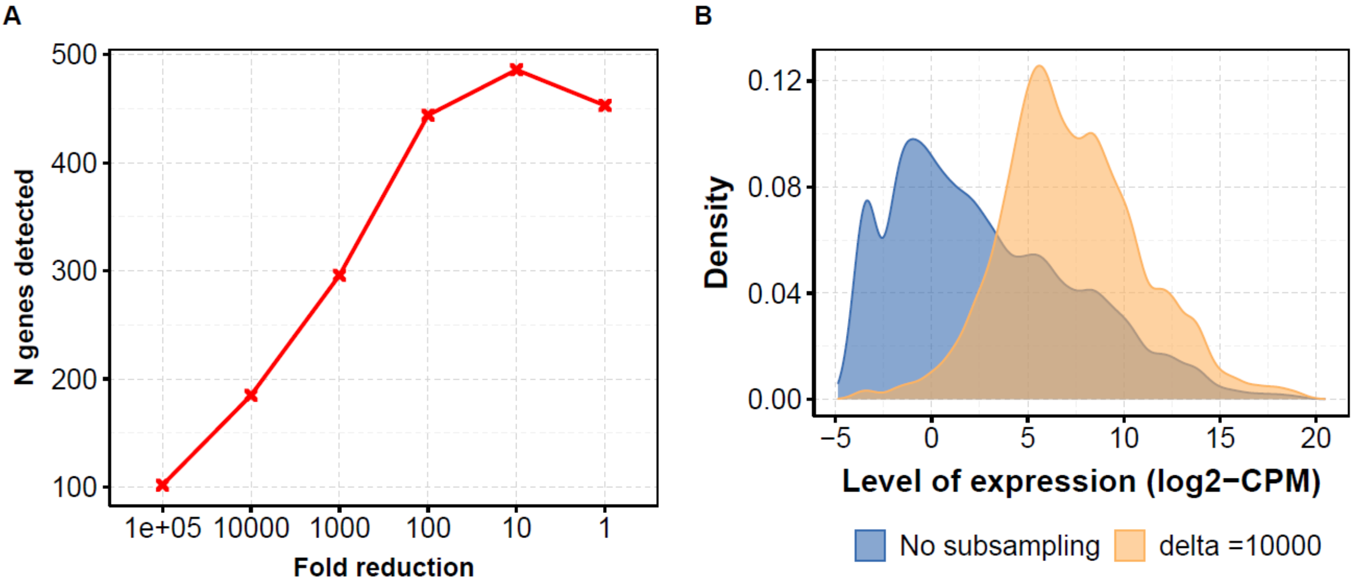

Supplementary Fig. S12. Number of genes (miRNAs) detected for different fold reduction (A) and level of expression (log2-CPM) of the genes detected without subsampling (blue) and after subsampling by a factor 10,000 for KIRP.

We defined the ‘genes detected’ as the miRNAs for which the count per million (CPM) data is higher than 1 for at least 1% of the patients.

Similar results are observed for other cancers and for mRNA-seq data (data not shown).

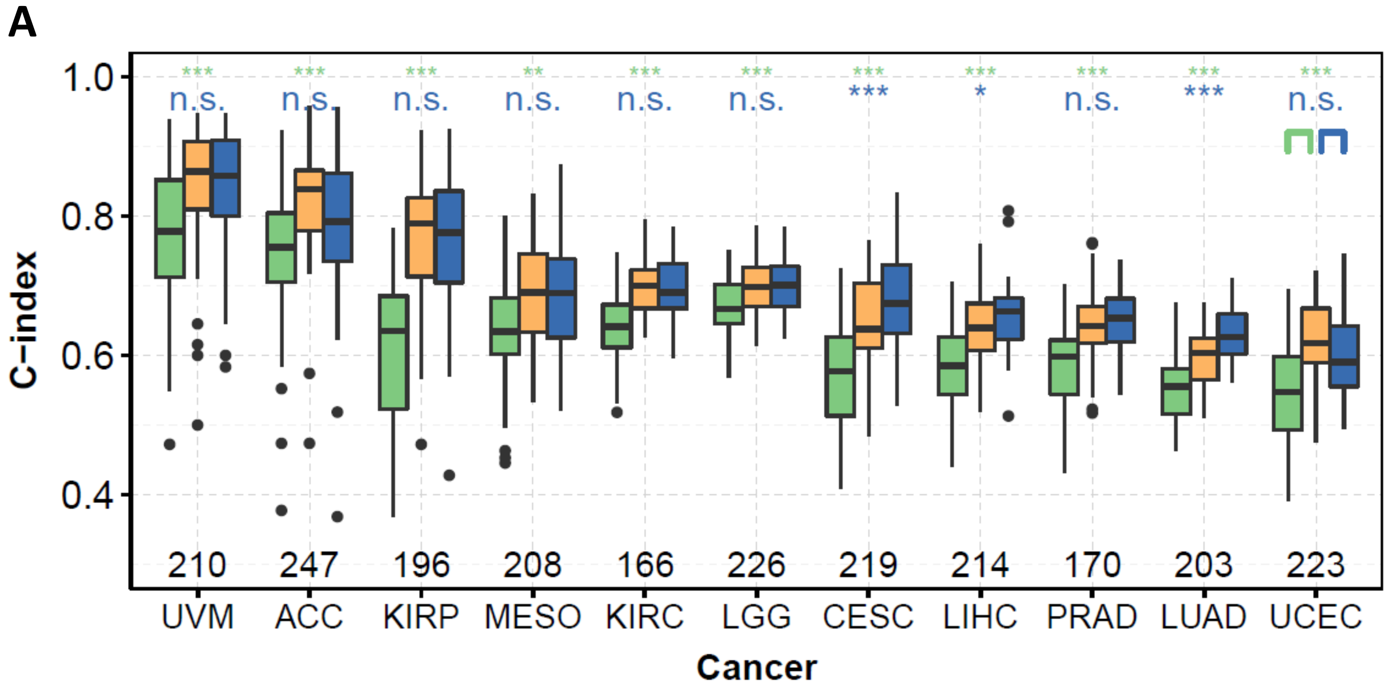

Supplementary Fig. S13. C-indices (A) and IBS (B) obtained after subsampling by a factor 10,000 (green), with the same miRNAs (~200 most expressed) but without subsampling (orange), and with all the miRNAs (~500) and without subsampling (blue) for the Cox model with elastic net penalty.

We computed the metrics by 10 repetitions of a K-fold cross-validation (K=5).

Above are the Benjamini-Hochberg corrected indication of the p-values obtained after a pairwise one-sided Wilcoxon test: green stars for orange versus green "scenario", and blue stars for blue versus orange "scenario".

***: $p \leq 0.001$, **: $p \leq 0.01$, *: $p \leq 0.05$, +: $p \leq 0.1$, n.s. : $p > 0.1$

We indicated the number of genes detected after subsampling by a factor 10,000 in black at the bottom of each graphics).

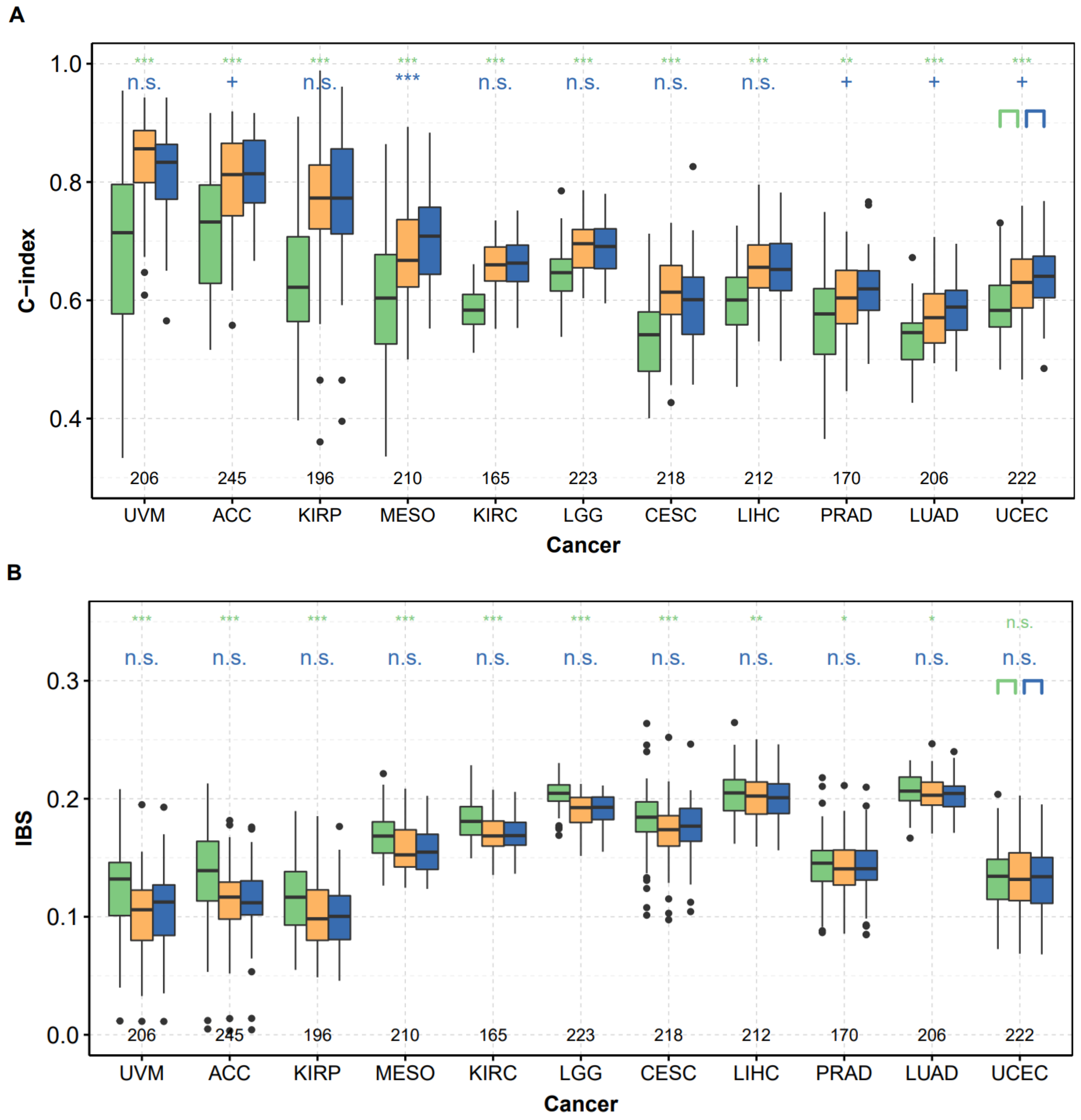

Supplementary Fig. S14. C-indices (A) and IBS (B) obtained after subsampling by a factor 10,000 (green), with the same miRNAs (~200 most expressed) but without subsampling (orange), and with all the miRNAs (~500) and without subsampling (blue) for random survival forest.

We computed the metrics by 10 repetitions of a K-fold cross-validation (K=5).

Above are the Benjamini-Hochberg corrected indication of the p-values obtained after a pairwise one-sided Wilcoxon test: green stars for orange versus green "scenario", and blue stars for blue versus orange "scenario".

***: p ≤ 0.001, **: p ≤ 0.01, *: p ≤ 0.05, +: p ≤ 0.1, n.s. : p > 0.1

We indicated the number of genes detected after subsampling by a factor 10,000 in black at the bottom of each graphics).

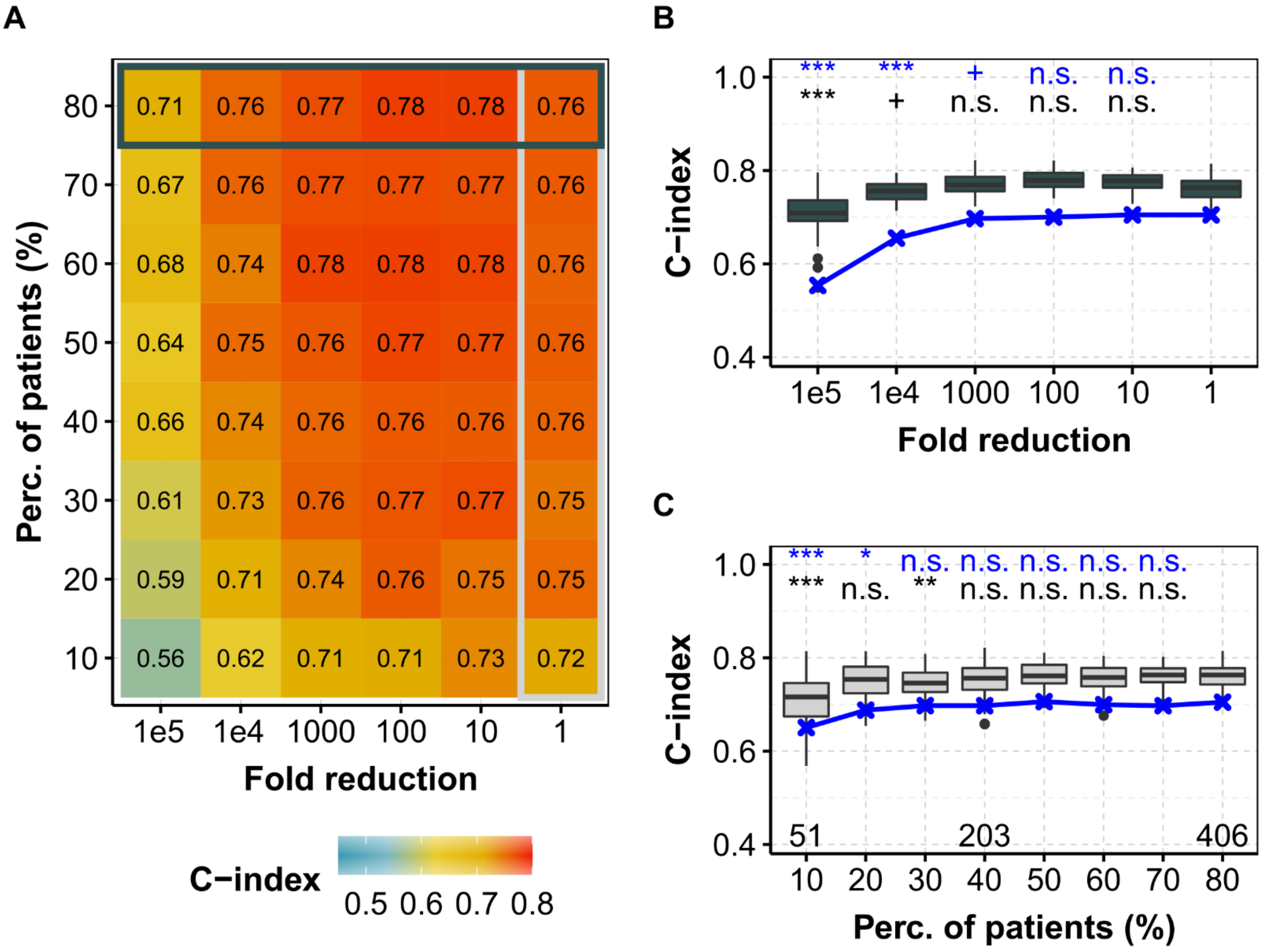

Supplementary Fig. S15. C-index obtained for different fold reduction factors and percentage of patients in the training dataset for KIRC (ccRCC, TCGA) with the Cox model, assessed on the E-MTAB-1980 dataset.
Same as Fig. 1 but for the independent E-MTAB-1980 dataset.

(A) Median C-index for different degradation of both sequencing depth (x axis) and percentage of patients (y axis) in the training dataset for miRNA-seq data. Horizontal box highlights the case where all of the 80% of patients are used and corresponds to (B), whereas vertical box focuses on the full available library size and corresponds to (C). **(B)** C-index for different fold reduction factors for miRNA-seq (gray boxplots) and mRNA-seq data (median values, in red) with 80% of the patients in the training dataset. Above is the p-value of a one-sided Wilcoxon test compared to no subsampling (*i.e.* $\delta = 1$). **(C)** C-index for different percentage of patients in the training dataset for miRNA-seq (light gray boxplots) and mRNA-seq data (median values, in red) with original TCGA sequencing depth. Above is the p-value of a one-sided Wilcoxon test compared to full dataset (i.e. 80%).
blue, mRNA-seq from TCGA; gray (boxplots), mRNA-seq from E-MTAB-1980.

In each case, we computed the C-indices by 10 repetitions of a 5-fold cross validation.
***: $p \leq 0.001$, **: $p \leq 0.01$, *: $p \leq 0.05$, +: $p \leq 0.1$, n.s. : $p > 0.1$

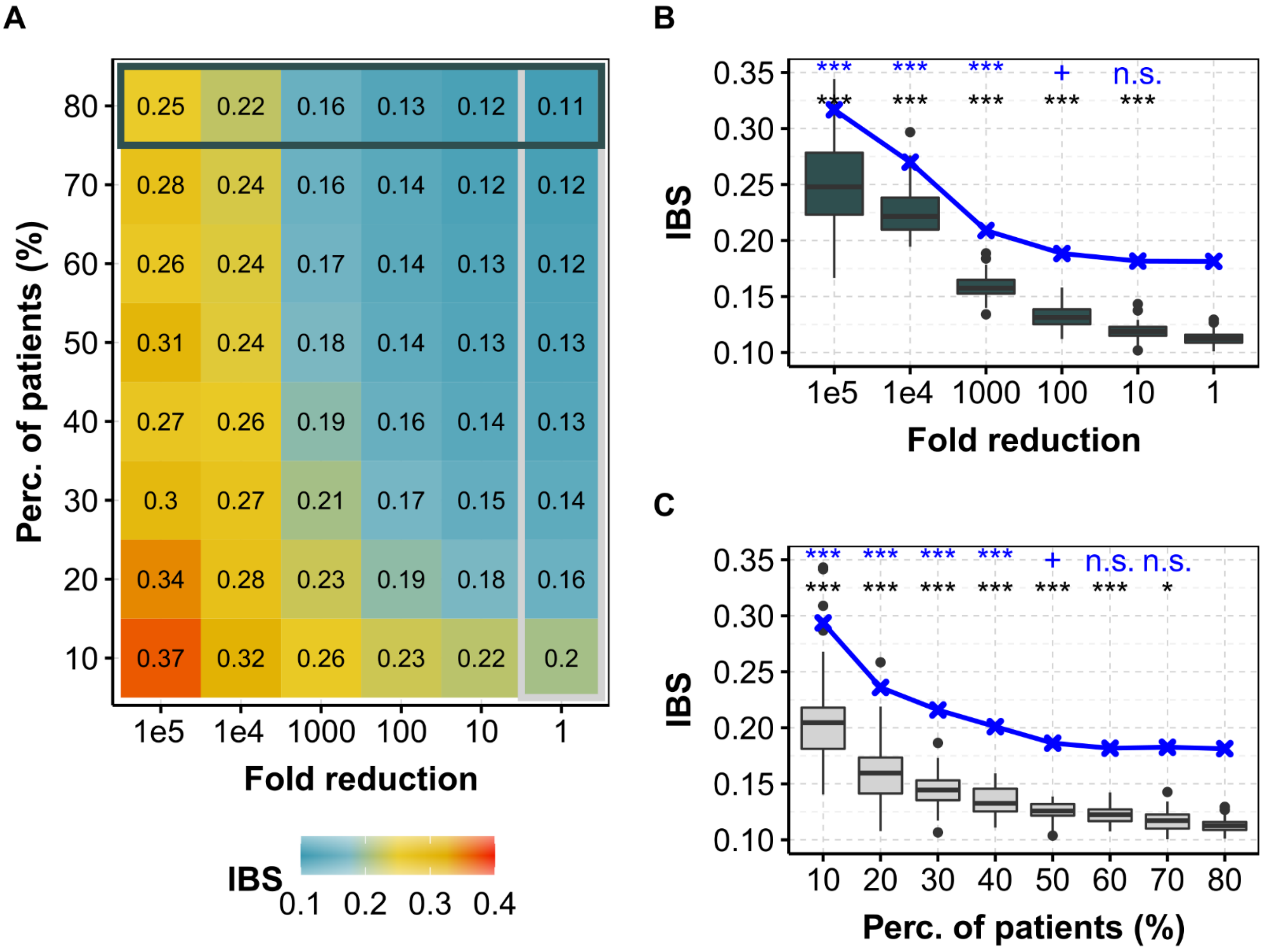

Supplementary Fig. S16. IBS obtained for different fold reduction factors and percentage of patients in the training dataset for KIRC (ccRCC, TCGA) with the Cox model, assessed on the E-MTAB-1980 dataset.

Same as Fig. 1 but for the independent E-MTAB-1980 dataset and for IBS.

(A) Median IBS for different degradation of both sequencing depth (x axis) and percentage of patients (y axis) in the training dataset for miRNA-seq data. Horizontal box highlights the case where all of the 80% of patients are used and corresponds to (B), whereas vertical box focuses on the full available library size and corresponds to (C). **(B)** IBS for different fold reduction factors for miRNA-seq (gray boxplots) and mRNA-seq data (median values, in red) with 80% of the patients in the training dataset. Above is the p-value of a one-sided Wilcoxon test compared to no subsampling (*i.e.* $\delta = 1$). **(C)** IBS for different percentage of patients in the training dataset for miRNA-seq (light gray boxplots) and mRNA-seq data (median values, in red) with original TCGA sequencing depth. Above is the p-value of a one-sided Wilcoxon test compared to full dataset (i.e. 80%).

blue, mRNA-seq from TCGA; gray (boxplots), mRNA-seq from E-MTAB-1980.

In each case, we computed the IBS values by 10 repetitions of a 5-fold cross validation.

***: $p \leq 0.001$, **: $p \leq 0.01$, *: $p \leq 0.05$, +: $p \leq 0.1$, n.s. : $p > 0.1$
